# Media Exposure and Health in Europe: Mediators and Moderators of Media Systems

**DOI:** 10.1007/s11205-015-0933-6

**Published:** 2015-03-25

**Authors:** Niels Blom, Reneé van der Zanden, Moniek Buijzen, Peer Scheepers

**Affiliations:** 1 Research Master Social and Cultural Sciences, Radboud University Nijmegen, Nijmegen, The Netherlands; 2 Radboud University Nijmegen, Nijmegen, The Netherlands

**Keywords:** General health, Traditional media exposure, Contemporary media exposure, Media systems, Cross-national research

## Abstract

This study examined media exposure as an explanatory factor for individual and cross-national differences in self-assessed general health. In studying media exposure, traditional media (television, radio, and newspapers) and contemporary media (internet) were separately considered. Aside from hypotheses about the relation between media exposure and general health, we also tested hypotheses regarding the mediating role of social isolation and mean world syndrome as well as the moderating role of different media systems across countries. Therefore, we used European Social Survey 2010, covering 25 European countries (n = 36,692). The results of our multilevel regression analyses indicated that exposure to television was negatively related to general health, whereas exposure to radio and newspapers were positively related to health. For contemporary media, findings indicated consistent positive relations between internet exposure and health across. Furthermore, limited support was found for the mediating role of social isolation and the mean world syndrome in the link between media exposure and health. Across media systems, findings for the relations between exposure to the various types of media and health proved to be robust.

## Introduction and Research Questions

Major overviews and empirical evidence have been provided to explain individual and cross-national differences in self-assessed health. These differences in general health have been found to be associated with socioeconomic status (Mackenbach et al. 2008), social capital (Huijts and Kraaykamp 2012), (spousal) educational attainment (Huijts et al. 2010a), marital status (Huijts and Kraaykamp 2011a), and religious involvement (Huijts and Kraaykamp 2011b). However, when looking at lifestyle factors, media exposure has been scarcely studied as an explanatory factor, even though most Europeans are exposed to media daily (European Commission 2012). Two main arguments are proposed as to why the relation regarding media and health should be studied. First, media exposure can affect health negatively, by displacing social and physical activities that are as such positively related to self-assessed general health (Brown and Walsh-Childers 2002; Huijts 2011; Nie and Erbring 2000). Second, media can play a positive educator role (Chapman et al. 2005; Nattinger et al. 1998), by providing information that enables people to choose healthier lifestyles (Kenkel 1991). Therefore, this study aims to contribute to contemporary insights on general health by taking media exposure into account.

The few studies about media exposure and health that have been conducted have several limitations, which we will address in this study. First, previous studies have neglected the link between media exposure and *general* health; previous studies were limited to specific aspects of health-related behavior, such as smoking or drinking alcohol (Gutschoven and Van den Bulck 2005; Hammermeister et al. 2005; Perry 2006) or depression or feelings of loneliness (Hammermeister et al. 2005; Schooler et al. 2006; Tiggemann 2003). Rather than addressing a more general measurement of self-assessed health, these previous studies all focused on media exposure and various specific health components. In the present study, we study self-assessed general health, which is in line with the World Health Organization’s (WHO) definition of health. Their definition of health incorporates physical, mental and social well-being (Huijts 2011; WHO 1946). Subjective self-assessed general health is not an objective measure of health; however, various studies show that it is a highly reliable predictor for mortality and morbidity, thus health (see Huijts 2011). Our more overall measurement of general health combines subjective self-evaluations of people’s individual general health (European Social Survey 2012) and is therefore the most valid proxy of the WHO’s definition of health.^1^

A second limitation is that multiple mediators have been proposed to explain the relation between media exposure and health at the individual level (e.g. Tiggemann 2003; Strasburger et al. 2010; Perry 2006). However, many of these explanations have not been tested simultaneously. Our aim is to gain a better understanding of the relation between media exposure and health. Therefore, we will set up an integrated theoretical model, based upon various theoretical aspects, namely social isolation and mean world syndrome, and study these different mediating explanations simultaneously.

A third limitation concerns a lack of comparisons of the relation between media exposure and self-assessed general health across a large number of countries. Previous studies addressed only one country (e.g. Gutschoven and Van den Bulck 2005; Schooler et al. 2006; Tiggemann 2003) or compared just a few (e.g. Curran et al. 2009; Gray et al. 2005). Therefore, it is impossible to assess (dis-) similarities on a larger scale. We set out to test the relation between media exposure and health in as many countries as possible, to gain more robust insights. In this respect, an international classification of media systems might provide insights in cross-national variations in the relation between media exposure and health. Therefore, Hallin and Mancini’s (2004) classification of media systems is introduced, distinguishing between three different systems (i.e. Mediterranean, North/Central European, and Liberal model), and add the Eastern European system, that potentially moderate relations between media exposure and health.

In order to address these limitations, we aim to answer the following research questions: (1) To what extent is media exposure associated with personal health in a cross-national perspective? (2) To what extent is the relation between media exposure and self-assessed general health (a) mediated by characteristics of social isolation and mean world syndrome and (b) moderated by different media systems?

Our research questions will be investigated using recent, high quality data from the European Social Survey (European Social Survey 2012), containing valid measurements of general health and media exposure. We apply a multilevel framework in which different individual and contextual characteristics are proposed and tested, enabling us to innovatively address the relation between media exposure and health in European societies.

## Theoretical Framework and Hypotheses

**Fig. 1:**
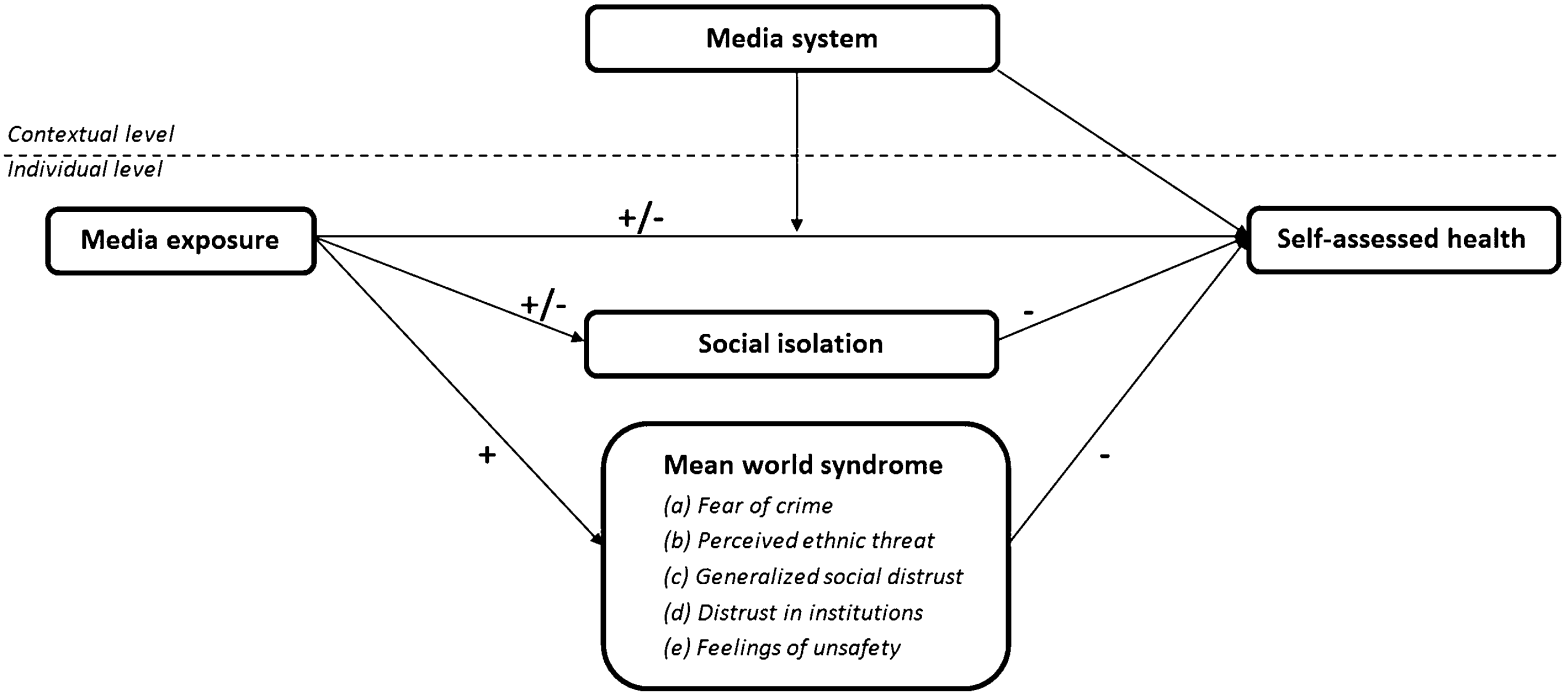
Integrated theoretical framework

In the following section, we formulate hypotheses based upon various previous theoretical and empirical insights that are set up in an integrated theoretical model. Here we will explain the relation between media exposure and self-assessed general health, provide mediators, and investigate possible moderations by media systems on these relations. This model is displayed visually in Fig. 1, and will be explained in more detail in three sections. First, the relation between media exposure and self-assessed health is discussed, in which media exposure displaces other activities and functions as educator. Second, the relation between media exposure and health with the mediating roles of social isolation and the mean world syndrome are discussed. Third, the moderating role of different media systems across countries is reviewed. As we set out to study the relation between media exposure and health, we distinguish between traditional media exposure (television, radio, and newspapers) and contemporary media exposure (internet).

### Media Exposure and Personal Health: Displacement of Other Activities and the Educating Role of Media

Traditional media channels are central providers of entertainment, providing an important leisure time activity (Vorderer et al. 2004). According to displacement theory, individuals only have a limited amount of leisure time available, posing constraints on their activities (Dutta-Bergman 2005; Nie and Erbring 2000). Because of time displacement, television exposure is related negatively with social and physical activities (Keim et al. 2004; Putnam 2001; Saelens et al. 2002; Brown and Walsh-Childers 2002). Considering that exposure to television and other types media are all time consuming, people who are exposed to media can spend less time on social and physical activities that are related to health (Huijts 2011; Huijts and Kraaykamp 2012). Therefore, we hypothesize that:

#### **Hypothesis 1a**

The more people are exposed to traditional media, the lower the level of self-assessed general health.

However, traditional media report on health related developments and explain health issues, providing cues about what issues should be at the forefront of people’s concerns (Brodie et al. 2003). Despite the negative influence of displacement, exposure to media may be beneficial to people’s health due to the educating role of media (Chapman et al. 2005; Nattinger et al. 1998). This is underpinned by the fact that more than half of the (American) public indicates that news is their most important source of health information (Brodie et al. 2003). Exposure to traditional media may therefore be of main importance in providing information on health issues (Brodie et al. 2003; Norris 1996). These health-related messages may shape people’s health-related behaviors, improving people’s knowledge on the relations between health behaviors and health outcomes and helps people choose healthier lifestyles (Strasburger et al. 2010; Brown and Walsh-Childers 2002; Kenkel 1991). Therefore, we hypothesize that:

#### **Hypothesis 1b**

The more people are exposed to traditional media, the higher the level of self-assessed general health.

Over the last decade, we expect that the educating role of exposure to internet to have become more dominant: seeking health information has become one of the most common activities on internet and as a result internet has become an increasingly important source of information (Brown and Walsh-Childers 2002; Greenberg et al. 2004). Even though medical researchers have expressed great concerns about the quality of health information on internet (e.g. Morahan-Martin 2004), findings amongst youngsters show that respondents recognize internet information that may not be credible and have strategies to test its reliability (Gray et al. 2005). Since knowledge provided by internet helps people to choose healthier lifestyles (Kenkel 1991), we hypothesize that:

#### **Hypothesis 1c**

The more people are exposed to internet, the higher the level of self-assessed general health.

### Exposure to Media and Health: Mediators

Many efforts have gone into studying the relation between media exposure and health, such as social isolation, and moreover, the mean world syndrome. In this study we examine the possible mediating effect of several determinants, starting with the mediating role of social isolation. We first discuss exposure to traditional media, followed by exposure to contemporary media.

#### Social Isolation

Putnam argued (1995, 2001), based on displacement theory, that exposure to television displaces time spent with social contacts, resulting in a decline of social capital in the United States and, vice versa, increasing people’s level of social isolation (Savelkoul et al. 2011). Since exposure to other types of traditional media such as radio and newspapers is as time consuming as television exposure, we expect these media types to displace social and leisure activities as well; therefore, increasing people’s level of social isolation. Studies have found negative relations between social isolation and health, due to increasing stress levels, less social support and less social control (see Huijts 2011; Huijts and Kraaykamp 2012). Hence, we derive the following hypothesis that:

##### **Hypothesis 2a**

The more people are exposed to traditional media, the more socially isolated they are, reducing self-assessed general health.

Contrary to the expected negative relation between media exposure and self-assessed health, Norris (1996) found some support for her claim that exposure to news and current affairs programs may be beneficial. Exposure to current affairs contributes positively to civic engagement and participation (Norris 1996). This in turn decreases people’s level of social isolation (Gelissen et al. 2012), which is beneficial to people’s level of general health (Huijts 2011). We assume that traditional media include news or current affairs. Therefore, we hypothesize that:

##### **Hypothesis 2b**

The more people are exposed to traditional media, the less socially isolated they are, inducing self-assessed general health.

In addition to exposure to these types of traditional media, exposure to contemporary media may relate to social isolation as well. First, information seeking on internet is related to a higher degree of civic participation, and thus to a lower level of social isolation (Moy et al. 2005; Shah et al. 2001). Second, internet is a way to connect with people, by means of social media (Ellison et al. 2007; Gil de Zúñiga et al. 2012; Steinfield et al. 2008) and email (Moy et al. 2005). Since social isolation is associated with lower levels of health, as discussed earlier (see Huijts 2011; Huijts and Kraaykamp 2012), our hypothesis reads that:

##### **Hypothesis 2c**

The more people are exposed to the internet, the less socially isolated they are, inducing self-assessed general health.

#### Mean World Syndrome

The mean world theory proposes that media exposure affects people’s worldview; that is media exposure induces a greater correspondence with the “media reality” as opposed to real world “facts” (Morgan et al. 2009). Research has primarily looked at the link of television exposure with worldviews, although previous studies also found that exposure to other forms of media exaggerated perceptions of reality as well (Dowler 2002; Heath and Gilbert 1996; Uslaner 2004).

Findings suggest that adjusted worldviews—in relation to media exposure—increase the fear of crime (Heath and Petraitis 1987) and ethnic threat (Ramasubramanian 2010), and decrease social trust (Shah 1998), trust in institutions (Cappella 2002) and feelings of safety (Gerbner et al. 1980). These factors in turn relate to people’s stress levels, as fear and anxiety interfere with coping strategies for alleviating stress (Solomon et al. 1991); and stress is, in turn, known to be related to lower levels of (general) health (Brosschot et al. 2006). Thus, negative world views induce peoples’ stress-level (the underlying mechanism), which is negatively related to health. Thus, we expect that all forms of media exposure increase people’s mean world syndrome. Therefore, our hypotheses read that:

##### **Hypothesis 3a–3e**

The more people are exposed to media, the higher the level of (a) fear of crime, (b) perceived ethnic threat, (c) generalized social distrust, (d) distrust in institutions, and (e) feelings of unsafety which in turn reduce people’s self-assessed general health.

### Exposure to Media and Health: Moderators

#### Media Systems

Previous studies have not been able to compare the relations between media exposure and self-assessed health across a large number of countries. In this study, we set out to test this relation across four different media systems. Due to established media policies, a link exists between the architecture of a media system and the media messages being sent (Curran et al. 2009). These systems are shaped by the wider political context as they are purposefully created and shaped by political interests (Freedman 2008; Hallin and Mancini 2004). One important aspect in which media systems differ greatly, is the extent to which they are commercialized. Based upon stable connections between media systems and political systems, Hallin and Mancini distinguished three media systems (2004), the *polarized pluralist model* (Mediterranean model), the *democratic corporatist model* (North/Central European model) and the *liberal model* (of Britain and Ireland).

The polarized pluralist model (of e.g. Portugal and Spain) is characterized by strong state intervention, as the state plays a large role as owner, regulator and funder of media, however, with limited capacity for effective regulation. The democratic corporatist model (of e.g. Scandinavian countries) is characterized by strong state intervention as well, but with protection of freedom of press, press subsidies and strong public-service broadcasting. The liberal model (of Britain and Ireland) has a strong tradition of public broadcasting, external pluralism and an early development of mass-circulation of commercial press (Hallin and Mancini 2004). The Eastern European, former communist countries have been disregarded so far. Nevertheless, we propose that their shared political history, state ownership of media institutions, and the related censorship of the media (Jakubowicz 1995) offer enough circumstantial evidence to take these countries into account as one group of former communist countries (Eastern European model).

In previous research, no indications for a relation between the different media systems and health have been presented. Therefore, in this study we will explore whether the relation between media exposure and general health is stable or varies across media systems, which would provide us with evidence on the moderating role of media systems.

## Method

### Data

To test our hypotheses, we used individual-level data from the European Social Survey (ESS) round 5, conducted in 2010/2011. Data were collected through face-to-face interviews held with individuals aged 15 and over, residing within private households, regardless of their nationality, citizenship, language or legal status. Samples are representative at a country level and in general response percentages are high; the overall response rate is above 60 percent (European Social Survey 2012). For more information about the data, see http://www.europeansocialsurvey.org.

The original dataset contains information on 50,781 respondents across 27 countries: Belgium, Bulgaria, Croatia, Cyprus, Czech Republic, Denmark, Estonia, Finland, France, Germany, Greece, Hungary, Ireland, Israel, Lithuania, the Netherlands, Norway, Poland, Portugal, Russian Federation, Slovakia, Slovenia, Spain, Sweden, Switzerland, the United Kingdom and Ukraine. Cyprus and Israel were left out of the analyses, for they do not fall into the typology of media systems, and there are no theoretical reasons to assume they do belong to one of the above mentioned types of media systems. Furthermore, only people between 25 and 75 years of age are included in the analyses, for there is possible health selection above the age of 75, and people younger than 25 have often yet to finish their education, possibly resulting in bias. After this age selection and listwise deletion of respondents with missing values on the individual characteristics, we estimated our models with 36,692 respondents across 25 European countries.

### Measurements

To measure *general health*, covering both physical and psychological health, respondents were asked: “how is your health in general?” The answer categories were: (1) “very bad”, (2) “bad”, (3) “fair”, (4) “good” and (5) “very good”. The categories are considered to be metric. Self-assessed health has been shown to be a reliable and valid measurement of health (Chandola and Jenkinson 2000; Lundberg and Manderbacka 1996). By using this measurement of self-assessed general health, we followed a prominent tradition in epidemiological research (Huijts and Kraaykamp 2011a). For the descriptive statistics (of all variables) we refer to Table 2 in the appendix.

To measure *media exposure*, four types of mass media exposure were assessed: television, radio, newspapers and internet. The first three items were measured with the questions “on an average weekday, how much time, in total, do you spend [watching television/listening to the radio/reading the newspapers]?” The answer categories range from (0) “no time at all” to (7) “more than 3 h”. These answer categories were considered to be continuous. Because a negative, yet nonlinear relation was found between radio and newspaper exposure with self-assessed health, we use dichotomous categories for both radio and newspapers exposure: (0) “no time at all”, versus (1) the other categories (see also the paragraph on linearity further on). For the use of internet, people were asked “how often do you use the internet, the World Wide Web or e-mail—whether at home or at work—for your personal use?” The answer categories ranged from (0) “no access at home or work” to (7) “every day”. These answer options constitute a metric scale, as will be explained at the end of this section.

*Social isolation* was measured with the single question: “How often do you meet socially with friends, relatives or work colleagues?” The answer categories being (1) “every day”, (2) “several times a week”, (3) “once a week”, (4) “several times a month”, (5) “once a month”, (6) “less than once a month” and (7) “never”. Previous studies used this variable to refer to the informal aspect of social capital (e.g. Savelkoul et al. 2011). We assessed the relation between these answer categories and the dependent variable to be linear, as will be shown at the end of this section.

*Fear of crime* was measured with the questions: “How often, if at all, do you worry about [becoming a victim of violent crime/your home being burgled]”. The answer categories are (1) “never”, (2) “just occasionally”, (3) “some of the time” and (4) “all or most of the time”. Although these items are often used as a part of a larger scale (Eitle and Taylor 2008; Taylor et al. 2009), several previous studies also relied on this selection of two items (Visser et al. 2013). The scale has been composed using Categorical Principle Component Analysis (CATPCA) in the 22nd SPSS edition.

The scale *perceived ethnic threat* was measured with three questions: “Would you say it is generally bad or good for [country]’s economy that people come to live here from other countries?”; “Would you say that [country]’s cultural life is generally undermined or enriched by people coming to live here from other countries?”; and “Is [country] made a worse or a better place to live by people coming to live here from other countries?” The answer categories ranged from 0 to 10. In line with previous studies (Coenders et al. 2004; Visser et al. 2013), we constructed a scale where we used the mean sum score (Cronbach’s α = 0.865). The scale was reversed, so a higher score meant people perceived more ethnic threat. This scale is equivalent across all countries of the ESS (Coenders et al. 2004); our principal factor analyses also pointed in this direction (lowest communality = 0.457, lowest factor loading = 0.676). The relation between these answer categories and the dependent variable were assessed as linear.

*Generalized social distrust* was measured with the questions: “Generally speaking, would you say that most people can be trusted, or that you cannot be too careful in dealing with people?”; “Do you think that most people would try to take advantage of you if they got the chance, or would they try to be fair?”; and “Would you say that most of the time people try to be helpful or that they are mostly looking out for themselves?” The answer categories ranged from 0 to 10. These questions are derived from the Rosenberg Trust Scale, which has been shown to be reliable and valid for the ESS countries (Cronbach’s α = 0.789) (Reeskens and Hooghe 2008). Our analyses showed that the factor solutions were comparable across countries. (the lowest communality was 0.159, the lowest factor loading 0.398. The other communalities were all above 0.200, the other factor loadings above 0.400.) We assessed the relation between the answer categories and the dependent variable as linear.

*Distrust in institutions* was measured using the questions how much confidence people have in politicians, parliament, the legal system, the police, the European Parliament and the United Nations. The answer categories ranged from (0) “no trust at all” to (10) “complete trust”. The scale was constructed by calculating the mean of the sum, and is subsequently reversed. It was reliable (Cronbach’s α = 0.912), valid and equivalent (Zmerli and Newton 2008; Zmerli et al. 2007). Principal factor analyses also showed that the factor solutions were roughly comparable across countries, although in eight countries two factors were found. Restricting the factors to one in these countries still provided sufficiently high communalities and factor loadings (lowest communality = 0.195, lowest factor loading = 0.446). The relation between the answer categories and the dependent variable were assessed as linear.

*Feelings of unsafety* were measured using the question “how safe do you—or would you—feel walking alone in this area after dark?”, with the answer categories ranging from (1) “very safe” to (4) “very unsafe”. Even though single-item indicators might be less reliable and valid, this straightforward question makes it unlikely for interpretation issues to arise (Visser et al. 2013). We assessed the relation between the answer categories and the dependent variables to be linear, as explained at the end of this section.

In the analyses, we controlled for *age* (in years), *age square*, *sex* (0 = male, 1 = female), *educational level,* *cohabiting status, religiosity* and *main activity*. The respondent’s highest completed level of education was assessed with multiple categories, ranging from (less than) lower secondary to higher tertiary. After testing for linearity, the categories were coded into three categories, namely: (less than) lower secondary, upper secondary and vocational and tertiary education. These categories constituted a linear relation with self-assessed health. The cohabiting status for all respondents was coded by the interviewers, stating if (1) “respondent lives with husband/wife/partner” or whether another situation is at place: (0) “all others”. Religiosity was measured by asking “[a]part from special occasions such as weddings and funerals, about how often do you attend religious services nowadays?” Answer categories ranged from (0) “never” to (7) “every day” and are considered continuous. The main activity of people was measured by asking “which of these descriptions best describes your situation (in the last 7 days)?” The answer categories were: “in paid work”, “in education”, “unemployed and actively looking for a job”, “unemployed, wanting a job but not actively looking for a job”, “permanently sick or disabled”, “retired”, “in community or military service”, “doing housework, looking after children or other persons” and “other”. The two unemployment categories were merged, for reasons of parsimony.

At the contextual level, we added one variable indicating the media system of one’s country. In line with Hallin and Mancini (2004), we distinguished four types of media systems: the polarized pluralist model (France, Greece, Portugal and Spain), the democratic corporatist model (Belgium, Denmark, Finland, Germany, Netherlands, Norway, Sweden and Switzerland) and the liberal model (Ireland and United Kingdom). The former communist model consists of the countries not classified by Hallin and Mancini (2004) (Bulgaria, Croatia, Czech Republic, Estonia, Hungary, Lithuania, Poland, Russia, Slovakia, Slovenia and Ukraine). Cyprus and Israel were left out of the analyses. We consider it important to note that Hallin and Mancini’s classification shows great similarities with the types of welfare state systems by Esping-Andersen (1990).

Finally, following Huijts (2011), we controlled for two macro-level control variables: *Gross domestic product (GDP) per capita* (in US dollars divided by 1000, adjusted for purchasing power parities) and *welfare state expenditure,* the percentage of the total health expenditure in a country that is covered by the government (World Health Organization, 2013). GDP per capita was retrieved from the United Nations Economic Commission for Europe (n.d.). Following Huijts (2011), we used the log linear function to account for the influence of very low and high income countries. Incorporating these macro-level controls avoids spurious relationships, for these controls are linked to general health and differ over media systems.

ANOVA-tests for (deviance from) linearity are conducted for the variables which we originally planned to include as metric variables in the analyses. The results of these tests are presented in Table 3 in the appendix. These analyses indicated that most relations are predominantly linear, even though there are also deviations from linearity. The added value of categorization of independent variables is limited (F-value of deviation from linearity differs <10 % of linear F-value), and therefore these will be included as linear indicators. The exceptions are media exposure to radio and newspaper and religious attendance, leading us to include the media exposure indicators as dummies (0 = no exposure, 1 = exposure), and include all religious attendance categories as dummies (reference is never attending religious services). All metric variables are grand-mean centered.

### Analytical Strategy

To account for the hierarchical structure of the data, multilevel linear regression analyses with the MIXED procedure were used in SPSS (IBM Corp, Armonk, NY) to produce more reliable parameter estimates and standard errors (Snijders and Bosker 1999). The intra-country correlations for our dependent variable general health indicated that 10.6 % (0.089/(0.089 + 0.749) × 100) of the variance was attributed to the country level, justifying the use of multilevel analysis. After the descriptive analyses, media exposure measures were taken into account in the first model (Table 1). Individual-level controls were added in the second model. In the third model, intermediating variables were added and in the final models the macro-level variables were incorporated. The parameters in these models contain unstandardized regression coefficients. Subsequently, tests for robustness have been conducted with different operationalizations of media exposure in the first robustness-check. In addition, checks for robustness of findings have been conducted in which self-assessed general health and the discussed mediators have been included as nominal or ordinal variables.

**Table 1:** Multi-level linear regression models for self-assessed general health (n = 36,692, N = 25)

	Model 0		Model 1		Model 2^a^		Model 3^a^		Model 4^a,b^	
	B	SD	B	SD	B	SD	B	SD	B	SD
*Media exposure*										
Television			−0.047***	0.002	−0.017***	0.002	−0.016***	0.002	−0.016***	0.002
Radio			0.105***	0.010	0.081***	0.010	0.070***	0.010	0.070***	0.010
Newspaper			0.031**	0.010	0.047***	0.009	0.031***	0.009	0.030***	0.009
Internet use			0.083***	0.002	0.026***	0.002	0.021***	0.002	0.021***	0.002
*Intermediating variables*										
Social isolation							−0.043***	0.003	−0.043***	0.003
Fear of crime							−0.067***	0.005	−0.067***	0.005
Ethnic threat							−0.010***	0.002	−0.010***	0.002
Social distrust							−0.031***	0.002	−0.031***	0.002
Distrust institutions							−0.019***	0.002	−0.019***	0.002
Feelings of unsafety							−0.070***	0.006	−0.070***	0.006
*Dummy’s institutions*										
Polarized pluralist (Mediterranean)									0.181	0.131
Democratic corporatist (Northern)									0.018	0.145
Liberal (UK & Ireland)									0.300	0.174
Former communist (Eastern)									ref.	
Intercept	3.705***	0.060	3.606***	0.050	2.893***	0.086	2.948***	0.083	2.890***	0.103
Individual-level variance	0.749	0.006	0.673	0.005	0.582	0.004	0.558	0.004	0.558	0.004
Country-level variance	0.089	0.025	0.061	0.017	0.072	0.021	0.062	0.018	0.032	0.009
−2 Log likelihood	93,675.733			89,731.998		84,398.308		82,819.132		82,802.442

*Source*: ESS 2010

* *p* < 0.05, ** *p* < 0.01, *** *p* < 0.001

^a^Controlled for education, sex, age, age square, cohabiting status, religious attendance and main daily activity

^b^Controlled for GDP (log) and government welfare expenditure

## Results

### Descriptive Results: Cross-National Differences in Media Exposure and Health

**Fig. 2:**
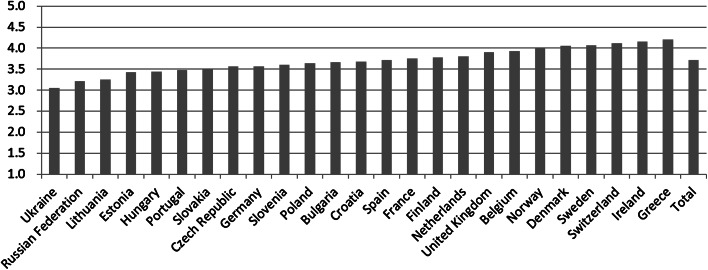
Self-rated general health by country (N = 36,692)

The levels of self-assessed general health differ greatly across countries, as can be seen in Fig. 2. The lowest average level of general health is found in Ukraine (3.069), the highest in Greece (4.200). Media exposure varies across countries as well, as shown in Table 4 in the Appendix. People in Switzerland report the lowest level of exposure to television (3.153), in Bulgaria the highest (5.558). Furthermore, people listen the most to the radio in Ireland (90.2 %), and the least in Bulgaria (40.2 %). Reading newspapers is most widespread in Norway, where 95.4 % of the population reads newspapers, followed by the Finnish and Swedish. Reading newspapers is least common in Greece, where 34.1 % reads newspapers. Lastly, internet exposure is especially prevalent in Scandinavia and the Netherlands, while it is less common in Eastern Europe. For the complete list of statistics of media exposure, see Table 4 in the Appendix.

### Media Exposure: Testing Hypotheses 1a–1c

We differentiated between exposure to traditional media (television, radio, and newspapers) versus contemporary media (internet). Two contradicting hypotheses were formulated for traditional media exposure, either emphasizing the displacement (hypothesis 1a), or educator effect (hypothesis 1b) of media exposure.

The results in Model 1 (Table 1) indicate that exposure to television is negatively related to general health, while exposure to the radio and newspapers is positively related to self-assessed general health. These results remain, although change slightly, in Model 2 when controlled for educational attainment, sex, age, age squared, religious attendance, cohabiting status, and main daily activity. Hence, these results indicate that for exposure to television, the displacement effect outweighs the educator effect; while on the contrary, for exposure to radio and newspapers the educator effect outweighs the displacement effect. Therefore, hypothesis 1a and 1b are both partly supported: hypothesis 1a is supported for exposure to television and hypothesis 1b is supported for exposure to radio and newspapers.

Next, exposure to contemporary media is explored. For contemporary media exposure we expected the educator effect to outweigh the displacement effect (hypothesis 1c). In model 2 we see that internet exposure indeed relates positively to general health, clearly supporting hypothesis 1c. There is positive relation between internet use and general health (b = 0.026). We can conclude that more frequent use of internet induces higher levels of general health, supporting hypothesis 1c.

### Exposure to Media and Health: Mediators

The next step in our study is adding all mediating variables to gain a better understanding of the relation between media exposure and general health. First, the correlations between the mediators, general health and media exposure are discussed. Next, we review the findings in relation to social isolation, separately for traditional and contemporary media. Hereafter, we discuss the mediating role of mean world syndrome.

As can be seen in Table 5 in the Appendix, the mediating variables social isolation and indicators of mean world syndrome are all related to media exposure and general health. Exposure to radio, news papers and internet are negatively linked to these mediators, meaning that a higher level of exposure to these types of media is associated with a lower degree of social isolation and a more positive world view. In contrast, exposure to television is positively related to (more) social isolation and a (more) negative world view. Furthermore, the mediators are negatively associated with general health, indicating that a stronger mean worldview and more social isolation are both associated with a lower level of self-assessed general health.

#### The Mediating role of Social Isolation: Testing Hypotheses 2a–2c

To test whether the relation between media exposure and self-assessed general health are mediated by social isolation (hypothesis 2a–2c), this mediating variable is included in model 3 in Table 1. The results indicate that social isolation indeed reduces people’s self-assessed general health (b = −0.043). However, including this determinant does not reduce the direct effect of media exposure on self-assessed general health. Therefore, we find only limited support for hypotheses 2a and 2b, since the reduction in the estimated parameters of exposure to traditional media, after a comparison of parameters in model 2 with parameters in model 3, is negligible. Also, we find no support for hypothesis 2c, because we do not find the expected reduction in the estimate of the parameter of internet use; after including social isolation in the models, the reduction of the parameters is negligible.

#### The Mediating role of the mean World Syndrome: Testing Hypotheses 3a–3e

To test whether the relations between media exposure and self-assessed health are mediated by indicators of the mean world syndrome (fear of crime, perceived ethnic threat, generalized social distrust, distrust in institutions, and feelings of unsafety, hypothesis 3a–3e), these mediating variables were included stepwisely. Because the media exposure estimates do not change substantially in these stepwisely built models, we only present the full-models. As can be seen in model 3 in Table 1, the mediating variables all relate negatively to self-assessed general health. Hence, we conclude that indicators of the mean world syndrome reduce self-assessed general health. We find, however, limited support for the hypotheses regarding the relation between traditional media exposure and the mean world syndrome and self-assessed health: we expected to find a reduction in the parameters between model 2 and 3 of traditional media exposure with the dependent variables: these are, however, very modest. In sum, we only find limited support for hypotheses 3a–3e: indicators of the mean world syndrome do relate to self-assessed health, but we only find limited support that this syndrome explains the relation between media exposure and health.

### Exposure to Media and Health: Moderators

#### Media Systems: Exploring the Relations Across Media Systems

To explore relations across different media systems, analyses were conducted with every media system as the reference category. Table 1, model 4 demonstrates that we found no significant differences between media systems in self-assessed health. Quite some country-level variance was explained by adding the macro-level characteristics to the models; the country-level variance is reduced by half in model 4 (in comparison to the previous models) for self-assessed general health.

To ascertain if estimates of media exposure and various intermediating variables are stable or vary across different media systems -to test for robustness- we re-calculated the estimates of model 2 and 3, but now separately for the four distinct systems (see Tables 6 and 7 in the Appendix). In comparing the estimated parameters of Table 1 across the distinct media systems, overall findings were found to be consistent and hence robust across media systems. However, we find some differences for the separate types of media.

Starting with television exposure, the pattern of relations between exposure and self-assessed general health in Table 1 is reproduced in Tables 6 and 7: exposure to television is negatively related to self-assessed general health. Most parameters are in the same direction in the different media systems, only one being non-significant (exposure to television in post-communist media systems). Second, exposure to the radio is relatively robust, being positively related to health in all media systems, except in liberal media systems where this difference is non-significant. Third, exposure to newspapers is positively related to self-assessed general health in the polarized pluralist, corporatist, and former communist media systems. The opposite relation has been found in the liberal media system. Lastly, internet exposure is positively related to self-assessed general health in all media systems.

Subsequently, looking at all mediating variables, we find relations with self-assessed health to be robust; all estimated parameters are negative and significant across the media systems. There is only one exception: ethnic threat which is non-significantly related to health in two media systems. Originally, we found limited support for the hypotheses regarding mediation of the relation between media exposure and self-assessed health. However, across media systems we find some support, especially for exposure to newspapers. With the findings of limited differences across media systems in the estimated parameters of media exposure as well as the mediating variables and health, our findings indicate that media systems do not moderate these relations.

### Media Exposure and Health: Testing for Robustness

As previously demonstrated, we found a link between media exposure and self-assessed health. To assess whether these findings are robust against variations in operationalizations, while accounting for small deviances from linearity, measurements on television and internet exposure were categorized in “low exposure” (‘no time at all’, ‘no access’ respectively), “high” (‘more than 3 h a day’, ‘every day’ respectively), and “medium” (the categories in between). The findings can be found in Table 8 in the Appendix. The results show substantial similarities to results discussed above. Both medium and high exposure to internet are related to a higher level of self-assessed general health, and high exposure to television is negatively related to self-assessed health. The parameter of a medium level of exposure to television is non-significantly related to self-assessed general health.

For the next tests of robustness, self-assessed general health is categorized in “bad health” (‘very bad’, ‘bad’ and ‘fair’ health) versus “good health” (‘good’ and ‘very good’ health). Next to the categorization of media exposure discussed above, the mediating variables were categorized as well (below versus above the grand mean). The check for robustness was conducted by using multi-level logistic regression analyses in the statistical package R by means of the glmmPQL procedure in the MASS package. The results in Table 9 in the Appendix again show substantial similarities with previous results; exposure to radio, newspaper, and internet are all positively related to self-assessed general health. The parameters for television exposure are, however, non-significant after taking the individual level controls into account. The mediators remain negatively related to self-assessed general health. Overall, these tests for robustness indicate that the findings for exposure to radio, newspapers, and internet are robust for varying operationalizations of self-assessed general health. However, the results regarding exposure to television and health require some attention, given the more measurement-sensitive results.

## Conclusion and Discussion

In this study, we set out to contribute to contemporary insights on the relation between media exposure and health. First, in contribution to previous studies, we looked at a more general measurement of health, namely self-assessed general health (e.g. Gutschoven and Van den Bulck 2005; Hammermeister et al. 2005; Schooler et al. 2006). This provided us with health measurements more in line with the WHO’s definition of health (Huijts 2011; World Health Organization 1946). Second, we simultaneously tested multiple mediating explanations of the relation between media exposure and self-assessed health to improve our understanding of the relation. Third, we contributed to the contemporary insights by using data from a large scope of different countries, which enabled us to look at cross-national variations in media exposure and health, as well as at differential effects of media systems.

We hypothesized, based on previous theoretical insights (Dutta-Bergman 2005), that media exposure displaces social and physical activities, which in turn may have detrimental or negative effects on health. We found, on the one hand, that exposure to television relate negatively to self-assessed general health. On the other hand, we found that exposure to radio and newspapers relate positively to self-assessed general health. Overall, these findings indicate that exposure to television may displace other activities outweighing possible education effects and vice versa, exposure to radio and newspaper makes educator effects outweigh displacement effects. We found that these relations are quite robust across different media systems.

Moreover, we expected frequent users of contemporary media (internet) to report higher levels of self-assessed general health: knowledge helps people to choose healthier lifestyles (Kenkel 1991), and searching for (health) information is one of the most common activities on the internet (Brown and Walsh-Childers 2002; Greenberg et al. 2004). Our findings support this hypothesis, evidenced by strong and consistent positive associations between internet exposure and general health. These relations are also robust across most media system. Because of this consistent finding, it is interesting for future research to take a closer look at internet use and health, and to distinguish between different types of content.

By simultaneously testing multiple intermediating explanations for the relation between traditional and contemporary media exposure and self-assessed general health, we introduced hypotheses derived from the social capital theory and the mean world theory. We found, in line with Huijts and Kraaykamp (2012), that social isolation actually relates negatively to general health. However, social isolation appeared not to contribute strongly to explain the relations between media exposure and health. Furthermore, a mean world view also appeared to reduce self-assessed health, but did also not contribute to explain the relation between media exposure and health. Therefore, our findings suggest that the relation between media exposure and self-assessed health is only marginally explained by these intermediating indicators of social isolation and the mean world syndrome.

To further our understanding, panel data could be considered to be collected in future research, to increase the insights in the causal mechanisms of the relation between media exposure and health. Furthermore, we encourage future research to investigate possible varying effects of media content on health, especially in a cross-national context. However, one should be cautious when investigating media content and health in a cross-national perspective, because of diverse qualifications of, for instance, current affairs programs and overlapping categories as with infotainment. Finally, future research may take other leisure activities into account, which have demonstrated to have impact on health, to contribute to the insights on the displacement argument.

In this study, progress has been made by addressing variations in the relations between exposure to media and health across media systems. In our analyses, we found that media systems, with macro-level controls, explained half of the country-level variation. Moreover, results indicated that there were not so much differential effects across the different media systems. Testing for robustness (by estimating models across the four systems separately), showed substantial similarities across media systems in the relation between media exposure, the intermediating variables, and self-assessed general health. In spite of the fact that media systems vary largely in architecture and messages, as well as in commercialization, individual-level relations between television exposure and health are negative, whereas relations between exposure to radio, newspapers, and internet and self-assessed general health are positive.

## Appendix

See Tables 2, 3, 4, 5, 6, 7, 8 and 9.

**Table 2:** Descriptive statistics before centering of data (n = 36,692)

	Minimum	Maximum	Mean	SD
*Media exposure*				
Television	0.00	7.00	4.419	2.038
Radio	0.00	1.00	0.738	0.440
Newspaper	0.00	1.00	0.683	0.465
Internet use	0.00	7.00	4.153	3.033
*Control variables*				
Educational attainment	1.00	3.00	1.979	0.697
Sex (female = 1)	0.00	1.00	0.541	0.498
Age	25.00	75.00	49.547	13.995
*Religious attendance*				
Every day	0.00	1.00	0.001	0.085
More than once a week	0.00	1.00	0.026	0.160
Once a week	0.00	1.00	0.110	0.313
At least once a month	0.00	1.00	0.111	0.314
Only on special holy days	0.00	1.00	0.228	0.420
Less often	0.00	1.00	0.193	0.394
Never	0.00	1.00	0.325	0.468
Cohabiting	0.00	1.00	0.667	0.471
*Main daily activity*				
Paid work	0.00	1.00	0.550	0.498
Education	0.00	1.00	0.015	0.121
Unemployed	0.00	1.00	0.076	0.265
Sick/disabled	0.00	1.00	0.027	0.162
Retired	0.00	1.00	0.241	0.428
Community/military service	0.00	1.00	0.000	0.020
House work	0.00	1.00	0.084	0.278
Other	0.00	1.00	0.007	0.083
*Intermediating variables*				
Social isolation	1.00	7.00	3.297	1.581
Fear of crime	−1.13	3.43	0.019	0.991
Ethnic threat	0.00	10.00	5.114	2.166
Social distrust	0.00	10.00	4.991	2.004
Distrust institutions	0.00	10.00	5.837	2.069
Feelings of unsafety	0.00	3.00	1.052	0.796
*Dummy’s institutions*				
Polarized pluralist (Mediterranean)	0.00	1.00	0.177	0.382
Democratic corporatist (Northern)	0.00	1.00	0.308	0.462
Liberal (UK & Ireland)	0.00	1.00	0.104	0.305
Former communist (Eastern)	0.00	1.00	0.411	0.492
*Macro control variables*				
GDP (log)	1.90	4.05	3.310	0.443
Government health expenditure	55.70	85.50	72.616	9.336

*Source*: ESS 2010

**Table 3:** Linearity and deviation from linearity, in relation to self-assessed general health

	Linearity		Deviation from linearity	
	F-value	R	F-value	Eta
*Media exposure*				
Television exposure	927.834***	−0.157	27.372***	0.170
Radio exposure	118.339***	0.056	89.324***	0.132
Newspaper exposure	2.328	0.008	10.480***	0.042
Internet use	4388.050***	0.326	15.139***	0.330
*Intermediating variables*				
Social isolation	1003.180***	−0.162	60.976***	0.186
Fear of crime	459.964***	−0.111	6.592***	0.128
Perceived ethnic threat	971.236***	−0.160	5.322***	0.177
Generalized social distrust	1236.832***	−0.180	2.101***	0.186
Distrust in institutions	1096.410***	−0.170	2.395***	0.199
Feelings of unsafety	1538.902***	−0.201	11.927***	0.202
*Control variables*				
Religious attendance	20.969***	−0.024	4.732***	0.035
Educational attainment (3-levels)	1457.596***	0.195	2.735	0.196

*Source*: ESS 2010

* *p* < 0.05, ** *p* < 0.01, *** *p* < 0.001

**Table 4:** Media exposure by country (before centering)

	N	Television exp.		Radio exp.		Newspaper		Internet use	
		Mean	SD	Mean	SD	Mean	SD	Mean	SD
Belgium	1302	4.305	1.947	0.856	0.352	0.571	0.495	4.916	2.746
Bulgaria	1770	5.558	1.730	0.420	0.494	0.568	0.496	2.476	3.043
Croatia	1120	4.250	1.973	0.757	0.429	0.725	0.447	3.217	2.935
Czech Republic	1874	5.066	1.860	0.796	0.403	0.680	0.467	3.908	3.053
Denmark	1231	4.347	1.912	0.846	0.361	0.752	0.432	5.931	2.143
Estonia	1334	4.696	2.034	0.813	0.390	0.775	0.418	4.798	2.783
Finland	1446	3.830	1.991	0.791	0.407	0.914	0.281	5.247	2.554
France	1345	4.245	2.065	0.801	0.400	0.583	0.493	4.763	2.895
Germany	2336	4.202	2.016	0.859	0.348	0.778	0.416	4.583	2.829
Greece	2129	4.890	2.057	0.637	0.481	0.341	0.474	2.849	2.941
Hungary	1184	4.182	2.064	0.720	0.449	0.724	0.447	3.623	3.184
Ireland	1968	4.731	2.021	0.902	0.297	0.757	0.429	4.550	2.861
Lithuania	1094	4.388	2.043	0.693	0.462	0.737	0.441	3.165	3.174
Netherlands	1502	4.496	2.004	0.791	0.407	0.746	0.435	5.914	2.085
Norway	1206	3.919	1.843	0.862	0.346	0.954	0.211	6.055	1.941
Poland	1237	4.031	1.975	0.760	0.427	0.645	0.479	4.044	2.974
Portugal	1555	4.502	1.954	0.591	0.492	0.536	0.499	2.776	2.998
Russian Federation	1771	4.954	1.989	0.425	0.494	0.572	0.495	2.710	3.084
Slovakia	1421	4.861	1.957	0.835	0.372	0.646	0.478	3.381	3.028
Slovenia	1039	3.625	1.947	0.856	0.352	0.802	0.399	4.167	2.942
Spain	1465	3.861	1.911	0.665	0.472	0.565	0.496	4.174	3.005
Sweden	1134	3.655	1.858	0.789	0.408	0.883	0.322	5.840	2.118
Switzerland	1146	3.153	1.909	0.824	0.381	0.880	0.325	5.101	2.610
Ukraine	1252	3.935	2.068	0.495	0.500	0.662	0.473	1.855	2.671
United Kingdom	1831	4.944	1.997	0.781	0.414	0.618	0.486	4.991	2.734
Total	36,692	4.419	2.038	0.738	0.440	0.683	0.465	4.153	3.033

*Source*: ESS 2010

**Table 5:** Correlation matrix between mediating variables, self-assessed general health and media exposure

	Self-assessed health^a^	Television exposure^a^	Radio exposure^b^	Newspaper exposure^b^	Internet use^a^
Social isolation	−0.162***	0.047***	−0.057***	−0.070***	−0.174***
Fear of crime	−0.111***	0.057***	−0.041***	−0.056***	−0.065***
Ethnic threat	−0.160***	0.139***	−0.070***	−0.109***	−0.247***
Social distrust	−0.180***	0.089***	−0.131***	−0.159***	−0.223***
Distrust institutions	−0.170***	0.065***	−0.138***	−0.174***	−0.229***
Feelings of unsafety	−0.201***	0.121***	−0.101***	−0.082***	−0.180***

*Source*: ESS 2010

* *p* < 0.05, ** *p* < 0.01, *** *p* < 0.001

^a^Pearson correlation coefficients

^b^Point-biserial correlation coefficients

**Table 6:** Multi-level linear regression models for self-assessed general health by media system (n = 36,692, N = 25)

	Polarized pluralist model				Democratic corporatist model			
	Model 2		Model 3		Model 2		Model 3	
	B	SD	B	SD	B	SD	B	SD
*Media exposure*								
Television	−0.014**	0.005	−0.016**	0.005	−0.035***	0.004	−0.029***	0.004
Radio	0.089***	0.021	0.077***	0.021	0.072***	0.019	0.061**	0.019
Newspaper	0.068***	0.020	0.047*	0.020	0.096***	0.019	0.069***	0.019
Internet use	0.009*	0.004	0.007	0.004	0.023***	0.003	0.016***	0.003
*Intermediating variables*								
Social isolation			−0.042***	0.006			−0.037***	0.005
Fear of crime			−0.057***	0.009			-0.048***	0.009
Ethnic threat			−0.005	0.005			−0.013**	0.004
Social distrust			−0.024***	0.006			−0.036***	0.005
Distrust institutions			−0.016**	0.006			−0.031***	0.005
Feelings of unsafety			−0.041**	0.013			−0.083***	0.011
Intercept	3.666***	0.195	3.765***	0.216	2.563***	0.142	2.513***	0.136
Individual-level variance	0.580	0.010	0.562	0.010	0.580	0.008	0.558	0.007
Country-level variance	0.050	0.036	0.087	0.062	0.025	0.013	0.017	0.009
−2 Log Likelihood	14,913.616		14,714.483		25,952.601		25,513.154	

*Source*: ESS 2010

* *p* < 0.05, ** *p* < 0.01, *** *p* < 0.001

Controlled for education, sex, age, age square, cohabiting status, religious attendance and main daily activity

**Table 7:** Multi-level linear regression models for self-assessed general health by media system (n = 36,692, N = 25)

	Liberal model				Former communist model			
	Model 2		Model 3		Model 2		Model 3	
	B	SD	B	SD	B	SD	B	SD
*Media exposure*								
Television	−0.028***	0.007	−0.027***	0.007	−0.003	0.003	−0.005	0.003
Radio	0.061	0.037	0.038	0.036	0.076***	0.014	0.070***	0.014
Newspaper	−0.079**	0.029	−0.081**	0.028	0.032*	0.013	0.022	0.013
Internet use	0.021***	0.006	0.016**	0.006	0.028***	0.003	0.025***	0.003
*Intermediating variables*								
Social isolation			−0.025**	0.008			−0.043***	0.004
Fear of crime			−0.074***	0.015			−0.076***	0.007
Ethnic threat			0.002	0.006			−0.012***	0.003
Social distrust			−0.048***	0.008			−0.024***	0.003
Distrust institutions			−0.023**	0.008			-0.017***	0.003
Feelings of unsafety			−0.078***	0.018			−0.081***	0.009
Intercept	3.854***	0.233	3.981***	0.231	2.873	0.115	2.949***	0.111
Individual-level variance	0.627	0.014	0.598	0.014	0.544	0.006	0.517	0.006
Country-level variance	0.010	0.010	0.011	0.011	0.037	0.016	0.032	0.014
−2 Log Likelihood	9012.583		8836.726		33,697.425		32,925.561	

*Source*: ESS 2010

* *p* < 0.005, ** *p* < 0.01, *** *p* < 0.001

Controlled for education, sex, age, age square, cohabiting status, religious attendance and main daily activity

**Table 8:** Multi-level linear regression models for self-assessed general health, categorized media exposure measurements (n = 36,692, N = 25)

	Model 1		Model 2^a^		Model 3^a^		Model 4^a,b^	
	B	SD	B	SD	B	SD	B	SD
*Media exposure*								
Television medium	−0.033	0.025	0.007	0.023	0.014	0.022	0.014	0.022
Television high	−0.240***	0.026	−0.076**	0.024	−0.063**	0.024	−0.062**	0.024
Radio exposure	0.100***	0.010	0.078***	0.010	0.067***	0.010	0.067***	0.010
Newspaper exposure	0.025*	0.010	0.043***	0.009	0.027***	0.009	0.027***	0.009
Internet medium	0.447***	0.012	0.164***	0.012	0.151***	0.012	0.150***	0.012
Internet high	0.558***	0.011	0.166***	0.012	0.137***	0.012	0.137***	0.012
*Intermediating variables*								
Social isolation					−0.043***	0.003	−0.043***	0.003
Fear of crime					−0.067***	0.005	−0.067***	0.005
Ethnic threat					−0.010***	0.002	−0.010***	0.002
Social distrust					−0.031***	0.002	−0.031***	0.002
Distrust institutions					−0.019***	0.002	−0.019***	0.002
Feelings of unsafety					−0.071***	0.006	−0.071***	0.006
*Dummy’s institutions*								
Polarized pluralist (Mediterranean)							0.184	0.131
Democratic corporatist (Northern)							0.020	0.145
Liberal (UK & Ireland)							0.299	0.174
Former communist (Eastern)							ref.	
Intercept	3.358***	0.056	2.784***	0.089	2.843	0.086	2.784	0.106
Individual-level variance	0.674	0.005	0.582	0.004	0.557	0.004	0.557	0.004
Country-level variance	0.062	0.018	0.073	0.021	0.062	0.018	0.032	0.009
−2 Log Likelihood	89,761.300		84,368.641		82,777.227		82,760.432	

*Source*: ESS 2010

* *p* < 0.05, ** *p* < 0.01, *** *p* < 0.001

^a^Controlled for education, sex, age, age square, cohabiting status, religious attendance and main daily activity

^b^Controlled for GDP (log) and government welfare expenditure

**Table 9:** Multi-level logistic regression models for self-assessed general health, categorized media exposure measurements and mediators (n = 36,692, N = 25)

	Model 1		Model 2^a^		Model 3^a^		Model 4^a,b^	
	B	SD	B	SD	B	SD	B	SD
*Media exposure*								
Television medium	−0.021	0.069	0.089	0.075	0.116	0.076	0.116	0.076
Television high	−0.504***	0.072	−0.124	0.079	−0.086	0.080	−0.084	0.080
Radio exposure	0.218***	0.028	0.174***	0.030	0.161***	0.030	0.160***	0.030
Newspaper exposure	0.015	0.027	0.069*	0.029	0.051	0.029	0.050	0.029
Internet medium	1.034***	0.032	0.429***	0.037	0.421***	0.037	0.419***	0.037
Internet high	1.291***	0.029	0.430***	0.036	0.397***	0.037	0.394***	0.037
*Intermediating variables*								
Social isolation (0–1)					−0.220***	0.027	−0.219***	0.027
Fear of crime (0–1)					−0.309***	0.028	−0.310***	0.028
Ethnic threat (0–1)					−0.135***	0.028	−0.135***	0.028
Social distrust (0–1)					−0.255***	0.029	−0.253***	0.029
Distrust institutions (0–1)					−0.205***	0.029	−0.203***	0.029
Feelings of unsafety (0–1)					−0.358***	0.032	−0.357***	0.032
*Dummy’s institutions*								
Polarized pluralist (Mediterranean)							0.563	0.339
Democratic corporatist (Northern)							0.229	0.376
Liberal (UK & Ireland)							0.818	0.452
Former communist (Eastern)							ref.	
Intercept	−0.282*	0.139	−2.119***	0.271	−1.429***	0.271	−1.658***	0.312

*Source*: ESS 2010

* *p* < 0.05, ** *p* < 0.01, *** *p* < 0.001

^a^Controlled for education, sex, age, age square, cohabiting status, religious attendance and main daily activity

^b^Controlled for GDP (log) and government welfare expenditure

## Footnotes

1 Note: from this point forward general health and health are used interchangeably throughout this paper and always refer to self-assessed general health.
